# Isotretinoin-Induced Inflammatory Bowel Disease: Is There a Real Association?

**DOI:** 10.7759/cureus.29825

**Published:** 2022-10-02

**Authors:** Mohammed A Miqdad, Lina Alatta, Alaa Abdelsamad, Sally Fouda, Akhila Sai Sree Cherukuri, Mohammed Eltanany, Fahad Albogami, Saleh Al Draiweesh, Nadir Abdelrahman

**Affiliations:** 1 Geriatrics, Michigan State University, East Lansing, USA; 2 Internal Medicine, Dr. Sulaiman Al Habib Medical Group, Khobar, SAU; 3 Clinical Research, Texas Health University and Health Science Center, Dallas, USA; 4 Gastroenterology, King Fahad Specialist Hospital, Dammam, SAU; 5 Family Medicine - Geriatrics, Michigan State University College of Human Medicine, East Lansing, USA

**Keywords:** vitamin a analogue, isotretinoin, crohn's disease, ulcerative colitis, inflammatory bowel disease

## Abstract

Inflammatory bowel disease (IBD) is an autoimmune inflammatory disorder that affects the gastrointestinal system with an annual increase in incidence and prevalence worldwide. While the precise cause behind IBD remains obscured, certain genetic susceptibilities, in addition to environmental factors, may trigger the stimulation of the immunoinflammatory system against the gastrointestinal system, eventually resulting in IBD. Furthermore, certain medications have been proposed to increase the risk of developing IBD, such as isotretinoin. IBD has been reported during the post-marketing phase of isotretinoin. Subsequently, IBD development was added as a potential gastrointestinal adverse effect of isotretinoin. This review article aims to evaluate the possible association between isotretinoin exposure and the development of inflammatory bowel disease. We enrolled 32 relevant studies, including case reports, case-control, and cohort studies. The results were critically analyzed and reviewed by independent authors to answer the research question and achieve the primary endpoint.

## Introduction and background

Inflammatory bowel disease (IBD) is a chronic autoimmune inflammatory disease that affects the gastrointestinal tract and is classified into Crohn's Disease (CD) and Ulcerative Colitis (UC) [1]. The inflammation of the intestinal mucosa is characterized by the influx of neutrophils and macrophages that produce cytokines, proteolytic enzymes, and free radicals, resulting in inflammation and ulceration. Although the cause of IBD remains unknown, studies have provided evidence that the pathogenesis is associated with genetic susceptibility of the host, intestinal microbiota, environmental factors, and immunological abnormalities [2].

Risk factors for IBD include a family history of CD or UC, former smoking (CD/UC), current smoking (CD), appendicectomy (CD), tonsillectomy (CD), meat and meat products (CD), vitamin D deficiency (UC), alteration of the gut microbiota, and psychological stress [2-5]. The remaining factors, including urban living, antibiotics, oral contraceptives, cesarean section, isotretinoin exposure, total energy, fat, cholesterol, fatty acids and their sub-classifications, eggs, and soft drinks intake, were also found to have some association with IBD in Western and Eastern populations [3]. The prevalence of IBD has increased globally with a remarkable increase in incidence in the East, given the chronic nature of the disease and the relatively low mortality rate, leading to an exponential rise in IBD cases worldwide [6]. For instance, the prevalence of IBD in Canada is 725 per 100,000 and is steadily rising with an average annual percentage change of +2.86%. Similarly, the IBD prevalence continues to rise in North America, reaching to 0.3% in the twenty-first century and is predicted to exceed 0.6% in the upcoming 10 years. In Europe, approximately 0.3% of the population are estimated to have been diagnosed with IBD, corresponding to a total of 2.5-3 million IBD patients [6].

Isotretinoin is a synthetic retinoid derived from vitamin A. It has been used successfully to treat patients with acne vulgaris (AV) by reducing sebum production and keratinization by reducing the sebaceous gland size and function [7-8]. To date, isotretinoin has been the mainstay of AV treatment since its Food and Drug Administration (FDA) approval in 1982 [9]. Isotretinoin’s side effects include growth retardation, abnormal healing, and depression; yet, they are rare and should not contraindicate drug use [10]. Nevertheless, isotretinoin has been controversially associated with the development of IBD [11]. While recent epidemiological studies and a meta-analysis suggest that isotretinoin does not increase the risk of UC, several lawsuits have been filed implying a correlation between isotretinoin and UC [12]. Important clinical questions inconsistently addressed in guidelines include the association of isotretinoin and IBD. These topics are preferred to be included in the guidelines' recommendations as they are often raised in daily clinical practice [7]. Table 1 summarises the pharmacological principles of isotretinoin [13].

**Table 1:** Isotretinoin Pharmacological Principles LFTs; Liver function tests, CPK; Creatinine phosphokinase, CBC; Complete blood count

Drug Class	Systemic retinoid
FDA Indication	Severe, resistant, nodular acne that is unresponsive to standard therapy
Non-FDA Indications	Moderate acne, cutaneous T-cell lymphoma, neuroblastoma, and prevention of squamous cell carcinoma in high-risk patients
Mechanism of action	Inhibits sebaceous gland function and keratinization
Administration	Orally in a capsule form
Absolute contraindications	Pregnancy, women who are planning to get pregnant, or those with hypersensitivity to vitamin A
Monitoring:	1. Pre-treatment: LFTs, lipid profile, blood glucose, CPK, and CBC. 2. Ongoing monitoring: LFTs and lipid profile every week until establishing a response to isotretinoin
Antidote	Nil

## Review

Method

This study is a review article in which relevant studies, including case reports, case-control, cohorts, and systematic reviews were selected. No randomized clinical trials have been found mostly due to the medico-legal obligations. We searched the PubMed database for relevant articles using the following Mesh words: Inflammatory bowel disease, ulcerative colitis, Crohn's disease, isotretinoin, and vitamin A analogue. To minimize bias, three independent authors approved and analyzed the studies and four authors reviewed and validated the results. Moreover, any disagreements concerning the eligibility of the publication were discussed among the researchers, and a consensus was met. The primary endpoint was the development of IBD secondary to isotretinoin exposure. The inclusion and exclusion criteria are outlined in Table 2.

**Table 2:** Inclusion and Exclusion Criteria

Inclusion criteria:	
1. Adult Population > 18 years	
2. Articles relevant to the research topic	
3. Published in the last 15 years	
4. Articles published in the English language	
Exclusion criteria:	
All other articles that did not have one or more of the inclusion criteria	

Discussion

Isotretinoin and the Immune System

A possible mechanism of isotretinoin-induced UC is the prevention of epithelial cell growth and the activation of T-cells. Another theory is that the T-cells activated by isotretinoin express the α4β7 and CCR9 receptors, which are crucial to the gastrointestinal system's inflammation process [12]. Retinoids inhibit the chemotaxis of neutrophils and monocyte and alter neutrophil profiles, generating reactive oxygen species [14]. Besides, isotretinoin suppresses superoxide anion generation, and the subsequent impaired accumulation and operation of neutrophils underlie the intestinal inflammatory process in chronic granulomatous disease, which has been involved in the pathophysiology of CD [14]. Hence, the innate luminal response to luminal bacteria is perhaps impaired in response to isotretinoin exposure, resulting in an excessive adaptive immune response similar to CD pathogenesis and symptomatic intestinal inflammation [14].

Moreover, several reports show that isotretinoin causes some significant diseases such as sacroiliitis by triggering inflammation [15]. Recent studies indicate that high-density lipoprotein (HDL) has an essential role in controlling the activation, adhesiveness, and inflammation of monocytes and the increase of progenitor cells, enabling the proliferation of monocytes [16]. Also, HDL molecules enhance vasorelaxation and increase endothelial nitric oxide synthase expression. Due to these properties of monocytes, they have a pro-inflammatory and pro-­oxidant effect, while HDL-C has just an opposite function [16]. Therefore, the increase in Monocyte/HDL Ratio (MHR) represents a good indicator of inflammation. As a result, the MHR level can be used as a highly specific, inexpensive, and easily accessible parameter for determining the clinical activity of the disease in patients with UC [16]. Accordingly, isotretinoin might potentially trigger the inflammatory process by altering the lipid profile (including HDL), resulting in IBD development.

Contrarily, retinoic acid, a form of vitamin A enhances the intestinal barrier function by increasing various tight junction protein expressions, such as occludin, claudin-1, claudin-4, and zonula occludens-1. Impaired intestinal barrier function has been implicated in the pathogenesis of IBD; consequently, isotretinoin might prevent IBD development instead of triggering it [17]. However, isotretinoin also provides pleiotropic effects, including natural killer T-cell stimulation, B-cell differentiation, disruption of glycoprotein synthesis and epithelial tissue growth, apoptosis, and alteration of cytokines [17].

Isotretinoin Exposure and the Risk of IBD; Evidence-Based

Several studies have investigated the possible association between isotretinoin exposure and IBD development. For instance, three patients who developed IBD six months after starting isotretinoin were reported by Passier et al.; two of them developed UC, and one had CD [18]. Likewise, Papaconstantinou et al. reported a young male patient diagnosed with UC eight months following isotretinoin exposure [12]. Notably, Bharmal and Anderson have reported isotretinoin-induced IBD exacerbation in a female patient who presented with UC flare-up 14 weeks following isotretinoin initiation. Subsequently, a flexible sigmoidoscopy showed severely active colitis with multiple superficial ulcers, which were not present in the initial phase [19]. On the contrary, Femia and Ann Vleugels concluded that isotretinoin does not seem to increase the risk of hospitalizations due to IBD exacerbation [20].

Furthermore, Crockett et al. performed a case-control study to evaluate the association between isotretinoin use and IBD development. The study concluded that UC is highly linked to a prior isotretinoin exposure (OR 4.36, 95% CI 1.97-9.66), and no correlation was found between CD and isotretinoin use (OR 0.68, 95% CI 0.28-1.68) [21]. Additionally, increasing the dose of isotretinoin was associated with an increased risk of UC (OR per 20 mg increase in dose: 1.50, 95% CI 1.08, 2.09) [21]. Compared to patients not exposed to isotretinoin, the risk of UC was higher in those exposed for more than two months (OR 5.63, 95% CI 2.10-15.03) [21]. Moreover, isotretinoin adverse effects reports were reviewed by the FDA between 1997 and 2002; IBD was reported in 85 cases with isotretinoin use [22]. According to the Naranjo ADR probability scale, four cases (5%) scored in the "highly probable" range for IBD to be caused by isotretinoin, 58 cases (68%) were "probable," 23 cases (27%) were "possible," and no cases were "doubtful." The study concluded that isotretinoin might be a triggering factor for IBD in a subgroup of patients [22].

On the other hand, Wright et al. compared the risk of IBD among isotretinoin exposed and unexposed AV patients; the retrospective cohort analysis of the study concluded that there was no significant difference in odds of developing IBD at one year between isotretinoin exposed and unexposed AV patients (aOR 1.40; 95% CI, 0.95-2.05). The study showed that IBD incidence among isotretinoin-exposed AV patients is very low, and the risk appears similar to unexposed patients with AV [23]. A controversial association between isotretinoin use and IBD has been reported by Al-Breiki et al.; two cases acquired gastrointestinal symptoms after initiating isotretinoin therapy and were diagnosed with IBD. Hence, the study recommended a gastroenterologist referral if a previously healthy individual developed gastrointestinal symptoms during isotretinoin treatment [24]. Similarly, Crockett et al. studied the casual association between isotretinoin usage and IBD, which concluded that there is no adequate proof to confirm or refuse whether the association between IBD and isotretinoin exists [17]. The latter was supported by Bendezú-García et al. [25].

Considerable observational studies found no association or dose-response relationship between isotretinoin use and IBD development. For instance, Etminan et al. included 2159 IBD cases (1056 with UC and 1103 with CD) and corresponded them with 43180 controls in a nested case-control study; IBD adjusted RR was 0.99 (95% CI, 0.52-1.90). The RRs for UC was 1.10 (95% CI, 0.44-2.70) and CD was 0.91 (0.37-2.25) [26]. The pooled RR for IBD for the five studies was 0.94 (95% CI, 0.65-1.36), concluding that using isotretinoin does not raise the risk of both UC and CD [26]. Correspondingly, isotretinoin was not greatly associated with an increased risk of IBD in a population-based retrospective cohort study [27]. Also, a similar finding was found in Lee et al.’s meta-analysis [28].

Regardless, although it is unrelated to the scope of this paper, a possible association between IBD and AV was proposed by Coughlin [11]. Interestingly, Rashtak et al.'s retrospective study concluded that the IBD risk might be less in isotretinoin-exposed individuals [29]. Likewise, Racine et al. concluded in a population-based case-control French nationwide study that isotretinoin is associated with a decreased CD risk [30]. Although isotretinoin may induce acute colitis and pan-enteritis, it is unlikely to cause chronic IBD [31-32].

After carefully reviewing the current data in the literature, it seems that there is no actual association between isotretinoin exposure and the risk of inflammatory bowel disease development. Also, due to the difficulties of conducting a randomized control trial to answer this question, our search only focused on the available observational studies. While case reports proposed a correlation between isotretinoin exposure and IBD development, most analytic studies (case-control and cohorts) found no significant association.

Limitations

The majority of the positively linked studies are case reports. No randomized control trials had been conducted to answer this question, up to our knowledge. Thus, the available data are only observational studies. One more limitation is that isotretinoin is mainly used by the teenage group, and that population is out of our inclusion criteria. Still, it is worth running a meta-analysis and including teenagers as they represent a remarkable sample size for isotretinoin exposure.

## Conclusions

In summary, we presume that there is no significant association between isotretinoin exposure and the risk of IBD. However, isotretinoin might trigger the development of UC (not CD) in individuals with high susceptibility to UC. The decreased risk of CD secondary to isotretinoin exposure is another interesting research question that needs further analysis in a different study. We recommend running a meta-analysis on the current available studies to clarify the association between isotretinoin exposure and the risk of IBD development.
